# GmFT2a and GmFT5a Redundantly and Differentially Regulate Flowering through Interaction with and Upregulation of the bZIP Transcription Factor GmFDL19 in Soybean

**DOI:** 10.1371/journal.pone.0097669

**Published:** 2014-05-20

**Authors:** Haiyang Nan, Dong Cao, Dayong Zhang, Ying Li, Sijia Lu, Lili Tang, Xiaohui Yuan, Baohui Liu, Fanjiang Kong

**Affiliations:** 1 The Key of Soybean Molecular Design Breeding, Northeast Institute of Geography and Agroecology, Chinese Academy of Sciences, Nangang District, Harbin, China; 2 University of Chinese Academy of Sciences, Beijing, China; 3 Institute of Biotechnology, Jiangsu Academy of Agricultural Sciences, Nanjing, China; 4 State Key Laboratory of Tree Genetics and Breeding, Northeast Forestry University, Harbin, China; Vrije Universiteit Amsterdam, Netherlands

## Abstract

*FLOWERING LOCUS T* (*FT*) is the key flowering integrator in Arabidopsis (*Arabidopsis thaliana*), and its homologs encode florigens in many plant species regardless of their photoperiodic response. Two FT homologs, GmFT2a and GmFT5a, are involved in photoperiod-regulated flowering and coordinately control flowering in soybean. However, the molecular and genetic understanding of the roles played by GmFT2a and GmFT5a in photoperiod-regulated flowering in soybean is very limited. In this study, we demonstrated that GmFT2a and GmFT5a were able to promote early flowering in soybean by overexpressing these two genes in the soybean cultivar Williams 82 under noninductive long-day (LD) conditions. The soybean homologs of several floral identity genes, such as *GmAP1, GmSOC1* and *GmLFY*, were significantly upregulated by GmFT2a and GmFT5a in a redundant and differential pattern. A bZIP transcription factor, GmFDL19, was identified as interacting with both GmFT2a and GmFT5a, and this interaction was confirmed by yeast two-hybridization and bimolecular fluorescence complementation (BiFC). The overexpression of *GmFDL19* in soybean caused early flowering, and the transcription levels of the flowering identity genes were also upregulated by GmFDL19, as was consistent with the upregulation of GmFT2a and GmFT5a. The transcription of *GmFDL19* was also induced by GmFT2a. The results of the electrophoretic mobility shift assay (EMSA) indicated that GmFDL19 was able to bind with the cis-elements in the promoter of *GmAP1a.* Taken together, our results suggest that GmFT2a and GmFT5a redundantly and differentially control photoperiod-regulated flowering in soybean through both physical interaction with and transcriptional upregulation of the bZIP transcription factor GmFDL19, thereby inducing the expression of floral identity genes.

## Introduction

Plants integrate various environmental signals, such as photoperiod and temperature, to ensure flowering under those conditions that optimize seed production. In *Arabidopsis thaliana* (Arabidopsis), multiple pathways converge on a small number of floral integrator genes, which include the floral promoters *FLOWERING LOCUS T (FT)* and *TWIN SISTER OF FT (TSF)*, to integrate photoperiod, temperature, vernalization, and light quality signaling [1]. FT and TSF are members of a family of proteins similar to the mammalian phosphatidylethanolamine-binding domain protein (PEBP) [2], [3]. In addition to the FT-like proteins, the plant PEBP family consists of two other phylogenetically distinct groups of proteins, the TERMINAL FLOWER 1 (TFL1)-like proteins and the MOTHER OF FT AND TFL (MFT)-like proteins [4]–[8]. FT and TSF act redundantly to promote flowering under long-day (LD) photoperiods[7], [9], [10]. Arabidopsis FT and TSF proteins produced in the phloem [7], [11] and are transported to the shoot apex, where they dimerize with the bZIP transcription factor FD to activate the expression of *SUPPRESSOR OF OVEREXPRESSION OF CONSTANS 1* (*SOC1*) [9], [12] and the floral meristem identity genes *APETALA1* (*AP1*) and *LEAFY* (*LFY*) [13], [14]. FT-like proteins from various species function in a manner similar to that of FT regarding the induction of flowering, transport in the phloem, and interaction with FD-like proteins [15]–[18], suggesting that this general mechanism is likely widely conserved across flowering plants. However, the rice FT ortholog Hd3a interacts with OsFD1 indirectly through a 14-3-3 protein to form a ternary trimer known as the florigen activation complex in the nuclei of the shoot apex, where it activates the expression of *OsMADS15*, an *AP1* homolog that regulates flowering [19], [20].

Soybean, *Glycine max* (L.) Merr., is basically a short-day (SD) plant: its flowering is induced when the day length becomes shorter than a critical length. Soybean is grown at a wide range of latitudes, from at least North 50° to South 35°, although the cultivation area of each soybean cultivar is restricted to a very narrow range of latitudes. This wide adaptability has most likely been generated by genetic diversity at a large number of the major genes and quantitative trait loci that control flowering behavior. Nine major genes, *E1* to *E8* and *J*, that control flowering time and maturity have been previously identified in soybean [21]–[28]. Among these genes, *E1* has been cloned using a map-based approach and is assumed to be a legume-specific transcription factor containing a putative nuclear localization signal and a B3 distantly related domain [29]; *E2* has been identified as an ortholog of the Arabidopsis *GIGANTEA* gene [30]; and *E3* and *E4* have been confirmed as *PHYA* homologs using map-based cloning [31] and a candidate gene approach [32], respectively. Many allelic variations occur at the *E1, E3* and *E4* loci, and their allelic combinations condition soybean flowering time, regulate preflowering and postflowering photoperiod responses, and contribute greatly to the wide adaptability of soybean [33], [34]. Two *FT* homologs, *GmFT2a* and *GmFT5a*, are involved in the transition to flowering and these two genes coordinately control flowering in soybean [35]. The maturity genes *E1, E2, E3* and *E4* downregulate *GmFT2a* and *GmFT5a* expression to delay flowering and maturation under LD conditions, suggesting that GmFT2a and GmFT5a are the soybean flowering integrators and major targets in the control of flowering [29], [30], [35], [36]. In addition, two *SOC1* homologs, *GmSOC1* and *GmSOC1-like*, have been molecular characterized in soybean: the overexpression of *GmSOC1* partially rescued the late-flowering phenotype of the *soc1-1* Arabidopsis mutant under LD conditions [37], while the overexpression of *GmSOC1-like* promoted flowering in *Lotus corniculatus* [38]. These results suggest that the two soybean *SOC1* homologs may function as floral activators in soybean. A soybean *AP1* homolog, *GmAP1*, has also been isolated in soybean and is specifically expressed in the flower, especially in the sepals and petals. This gene caused early flowering and the alteration of floral organ patterns when ectopically expressed in tobacco [39]. Despite the economic importance of soybean, knowledge regarding its molecular mechanisms of flowering remains limited. Here, we report that the overexpression of *GmFT2a* and *GmFT5a* in soybean can promote early flowering by activating the expression of floral identity gene homologs such as *GmAP1*, *GmLFY* and *GmSOC1*. The GmFT2a and GmFT5a proteins interact with the bZIP transcription factor GmFDL19. The overexpression of *GmFDL19* in soybean can also induce the expression of floral identity genes. Additionally, we show that the bZIP transcription factor GmFDL19 is able to bind with the ACGT cis-element of the *GmAP1* promoter. Our results suggest that the putative flowering model FT/FD-AP1 is well conserved in the legume soybean and that GmFDL19 may act as the key component in the photoperiod-regulated flowering pathway controlled by GmFT2a and GmFT5a.

## Materials and Methods

### Plant materials and growth conditions

The soybean cultivars Harosoy, Williams 82 and Dongnong 50 were used in this study. All plants were grown in a growth chamber (Conviron ADAPTIS-A1000, Canada) at a consistent temperature of 25°C and an average photon flux of 300 µmol m^−2^s^−1^, supplied by T5 fluorescent lamps. Day length regimes were 12L/12D for SD and 16L/8D or 18L/6D for LD. Tissue-specific expression was analyzed using the cultivar Harosoy grown under SD. Total RNA was isolated from trifoliate leaves, shoot apices, roots, flowers, flower buds, and roots. For the temporal expression analysis, pieces of young, fully developed trifoliate leaves and shoot apices were bulk sampled at 4 hours after dawn from 4 individual plants grown under SD every five days from 10 DAE until 25 DAE. The trifoliate leaves and shoot apices from 4 plants of both transgenic and untransformed lines were bulk sampled at 4 hours after dawn at 20 DAE under the LD condition and stored until total RNA extraction.

#### Soybean genetic transformation

The cDNA sequences of Harosoy *GmFT2a*/*5a* and *GmFDL19* were first cloned into the pEASY-T1 vector (Transgene, Beijing, China). XbaI/SacΙ-digested fragments were then inserted at multiple cloning sites in the pTF101.1 vector, and the transgenes were driven by the cauliflower mosaic virus 35S promoter [40]. The *GmFT2a*/*5a*-pTF101 and *GmFDL19*-pTF101 constructs were used to transform the cultivars Williams 82 and Dongnong 50, respectively, following the cotyledonary node method [41]. T0, T1 and T2 transformants were screened by daubing 160 mg/L glufosinate into the preliminary leaves of the seedlings. Herbicide-resistant T2 plants were subjected to molecular and phenotypic analysis.

### RT-PCR and quantitative RT-PCR analyses

Total RNA was isolated and cDNA was synthesized as described in Kong et al. [35]. RT-PCR of *GmFDL19*, *GmAP1* (*a*, *b, c*, *d*), *GmSOC1a*, *GmSOC1b*, *GmLFY1, GmLFY2* and *Tubulin* (as an internal control) was conducted using cDNAs synthesized from total RNA. PCR conditions were as follow: one cycle of 5 min at 94°C; 30 cycles of 30 sec at 94°C, 30 sec at 55°C to 60°C (depending on the gene), and 30 sec at 72°C; and a final extension of 10 min at 72°C. RT-PCR was performed using homolog-specific primers to easily separate the RT-PCR products (approximately 500 bp) from the fragments amplified from genomic DNAs (>1 kb). The RT-PCR products were separated by electrophoresis in a 1% agarose gel and visualized with EtBr under UV light. Quantitative RT-PCR was performed as described in [35]. The quantitative RT-PCR mixture was prepared by mixing a 1 µl aliquot of the reaction mixture from the cDNA synthesis, 5 µl of 1.2 µM primer premix, 10 µl SYBR Premix ExTaq Perfect Real Time (TaKaRa Bio), and water to a final volume of 20 µl. The analysis was conducted using the DNA Engine Opticon 2 System (Bio-Rad). The PCR cycling conditions were as follow: 95°C for 10 sec, 55°C to 60°C (depending on the gene) for 20 sec, 72°C for 20 sec, and 78°C for 2 sec. This cycle was repeated 40 times. Fluorescence quantification was conducted before and after the incubation at 78°C to monitor the formation of primer dimers. The mRNA level of the *Tubulin* gene was used as a control for the analysis. A reaction mixture without reverse transcriptase was also used as a control to confirm that no amplification occurred from genomic DNA contaminants in the RNA sample. In all of the PCR experiments, the amplification of a single DNA species was confirmed using both melting curve analysis of the quantitative PCR and gel electrophoresis of the PCR products. The primers used for qRT-PCR and RT-PCR are listed in Table S1 and Table S2, respectively.

#### Identification of soybean *FD, AP1, LFY* and *SOC1* homologs

The database used for these searches is available at Phytozome (http://www.phytozome.net/soybean). Starting with the Arabidopsis FD, AP1, LFY and SOC1 protein sequences, TBLASTN searches were conducted against the soybean (*Glycine max*) gene index (release 1.0). The top 18 *FD*-like gene sequences producing high-scoring segment pairs were chosen and investigated further. Primers were designed to amplify cDNAs for each of the top 18 *FD*-like genes (Table S2). Seven pairs of full-length CDS PCR (Table S3) primers were designed to amplify the seven expressed *FD*-Like genes, and restriction sites (underlined) were included in the oligos to facilitate the cloning of the PCR products into the yeast vector pGADT7. A multiple sequence alignment and a neighbor-joining phylogenetic tree were constructed using DDBJ (http://clustalw.ddbj.nig.ac.jp/) online ClustalW software and Treeview 2.0. The tree was based on the full-length amino acid region including the bZIP domain and the SAP motif (Figure S1). The bootstrap percentage supports are indicated at the branches of the tree.

### Yeast two-hybridization assays

The yeast cloning vectors pGBKT7 and pGADT7, the control vectors pGADT7-T and pGBKT7-53, and the yeast strain Y2H used in the yeast-two hybridization assays were obtained from the Clontech company (http://www.clontech.com/). The yeast two-hybridization assays were performed according to the manufacturer's instructions. Soybean full-length CDS of *GmFT2a* and *GmFT5a* were inserted into pGBKT7 vectors to generate fused GAL4 DNA binding domains as the soybean baits. Full-length CDS of the three soybean *FD*-like genes containing the SAP motif (*GmFDL19*, *GmFDL08* and *GmFDL15*) were cloned into pGADT7 to generate fused GAL4 DNA activation domains as the soybean preys, and the full-length CDS of Arabidopsis *FD* was also cloned into pGADT7 to generate a positive control. Table S4 lists the primers and restriction sites used to generate the yeast bait and prey constructs. The bait and prey plasmids were cotransformed into the yeast strain Y2H using the lithium acetate method and selected on SD medium lacking leucine (Leu) and tryptophan (Trp). After 4 days of incubation at 30°C, the yeast cells were replated on selection plates with SD medium lacking Leu, Trp, histidine (His) and adenine (Ade) but including the X-α-gal substrate for the interaction test.

### Bimolecular fluorescence complementation

The full-length CDS of *GmFT2a* and *GmFT5a* were amplified using the primer pairs *GmFT2a*-NE-F/R and *GmFT5a*-NE-F/R, respectively (Table S5), and were then inserted into the pUC_SPYNE [42] vector, which contains the DNA encoding the N-terminus of YFP. The full-length cDNAs of *GmFDL08, GmFDL15, GmFDL19* and *FD* were amplified using the primer pairs listed in Table S5 and then inserted into the pUC_SPYCE [42] vector, which contains the DNA encoding the C-terminus of YFP. The recombined pUC_SPYNE/CE plasmids were cotransformed into Arabidopsis protoplasts using polyethylene glycol–mediated transfection, as described previously [43]. YFP-dependent fluorescence was detected 24 h after transfection using a confocal laser-scanning microscope (Zeiss LSM 510 Meta).

### Electrophoretic mobility shift assay

The full-length coding region of *GmFDL19* was amplified by PCR using the primer pair *GmFDL19*-29b-F/R (Table S6). The PCR product and the pET29b plasmid (Novagene, WI, USA) were digested with *NdeI* and *SalI*. After ligation, the construct was transformed into the *E. coli* competent cell line BL21 (DE3) (Transgene, Beijing, China) according to the manufacturer's instructions. The recombinant GmFDL19 protein was purified using the His tag purification nickel ion system (Kangweishiji, Beijing, China). EMSA was conducted using the recombinant GmFDL19 protein and the DNA products of the *GmAP1a* promoter obtained by hybridizing the forward and reverse complementary oligos containing the ACGT core sequence (Table S6). The EMSA assay was conducted using the EMSA kit (Invitrogen, www.Invitrogen.com, Cat #E33075). The DNA-protein complex samples were loaded into a TBE gradient 6% polyacrylamide native gel (Bio-Rad Laboratories, www.bio-rad.com) at 200 V for 45 minutes. The DNA in the gel was stained using SYBR Green, provided in the same kit, and visualized using the GE Typhoon LFA 9500 Imaging System (GE Healthcare Life Science, USA).

## Results

### Overexpression of *GmFT2a* and *GmFT5a* causes precocious flowering in soybean

**Figure 1:**
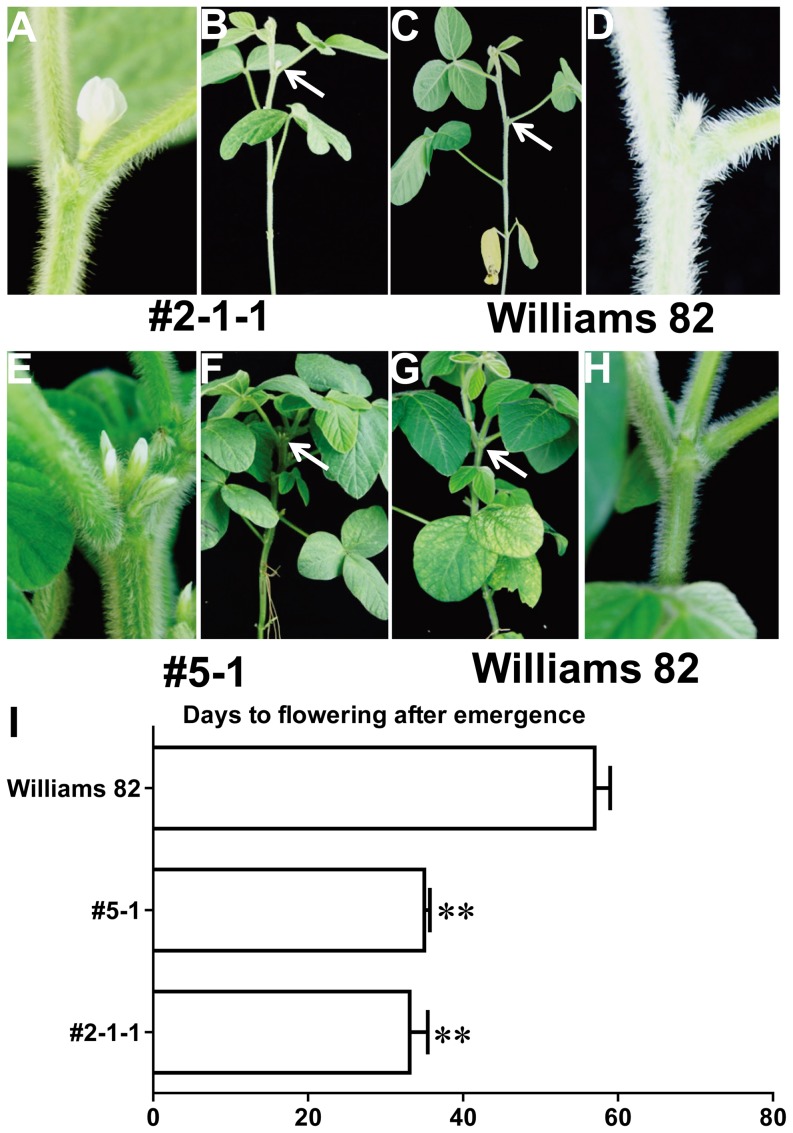
Overexpression of *GmFT2a* and *GmFT5a* causes precocious flowering in the soybean cultivar Williams 82. (A) The close shot of the transgenic plant in (B) shows the precocious flowers at the axils of the trifoliate leaves. (B) A transgenic *GmFT2a* plant showing precocious flowering at the axils of the trifoliate leaves. (C) A wild-type Williams 82 plant. (D) The close shot of the wild type Williams 82 plant in (C) does not show flowers at the axils of the trifoliate leaves. (E) The close shot of the transgenic plant in (F) shows the precocious flowers at the axils of the trifoliate leaves (F) A transgenic *GmFT5a* plant showing precocious flowering at the axils of the trifoliate leaves. (G) A wild-type Williams 82 plant. (H) The close shot of the wild-type Williams 82 plant in (G) does not show flowers at the axils of the trifoliate leaves. (I) Days to flowering from the emergence of the transgenic plants and wild-type plants. Averages and standard errors are calculated from four T2 plants for each construct and 5 Williams 82 plants. Double asterisks indicate significant differences from the corresponding wild-type Williams 82 at *P*<0.01.

Two *FT* homologs, *GmFT2a* and *GmFT5a*, are involved in photoperiod-regulated flowering, and these two genes coordinately control flowering in soybean [35]. To determine how these two *FT* homologs regulate soybean flowering, *GmFT2a* and *GmFT5a* were genetically transformed into the soybean cultivar Williams 82 under the control of the cauliflower mosaic virus (CaMV) 35S promoter. The overexpression of *GmFT2a* and *GmFT5a* caused the early flowering of Williams 82 even under noninductive LD (16L/8D) conditions (Figure 1A, B, C, D and Figure 1E, F, G, H). The transgenic *GmFT2a* T2 overexpression line #2-1-1 flowered at approximately 33 days after emergence (DAE) and the transgenic *GmFT5a* T2 overexpression line #5-1 flowered at approximately 35 DAE; however, the untransformed Williams 82 flowered at approximately 57 DAE (Figure 1I). These data suggested that both *GmFT2a* and *GmFT5a* are able to induce early flowering in soybean under noninductive LD conditions.

### GmFT2a and GmFT5a upregulate floral meristem identity genes

**Figure 2:**
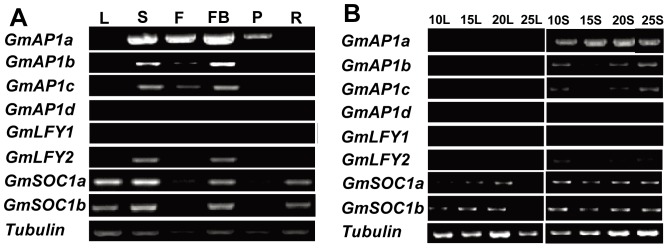
Temporal and spatial expression of soybean flowering-related genes. (A) Transcript levels of eight soybean flowering-related genes (*GmAP1a*, *GmAP1b*, *GmAP1c*, *GmAP1d*, *GmSOC1a*, *GmSOC1b*, *GmLFY1*, *GmLFY2*) in leaves and shoot apices of the soybean cultivar Harosoy under SD (12L/12D) conditions; *Tubulin* is included as an endogenous control. Samples were collected from 10 DAE to 25 DAE. DAE: days after emergence. L: leaves; S: shoot apex. (B) Tissue-specific expression analyses of eight flowering-related genes by RT-PCR under SD (12L/12D) conditions. L: leaves, S: shoot apices, F: flowers, FB: flower buds, P: pods, R: roots.

All flowering pathways converge onto floral integrators, including *FT* and *SOC1*, and induce the expression of floral meristem identity genes, including *AP1* and *LFY* [44], [45]. Several genes involved in the determination of flowering time have recently been isolated and characterized in soybean, including *GmAP1*, *GmSOC1*, *GmSOC1-like* and *GmLFY* [38], [39], [46]. By searching the soybean reference genome using Phytozome (http://www.phytozome.net/soybean), four *AP1* homologs were identified and designated as *GmAP1b* (Glyma01g08150), *GmAP1c* (Glyma08g36380), *GmAP1d* (Glyma02g13420) and *GmAP1a* (Glyma16g13070, *GmAP1*), the last of which has been characterized previously [39]. Spatial RT-PCR analyses suggested that *GmAP1a*, *GmAP1b* and *GmAP1c* were mainly transcribed in reproductive organs such as shoot apices, flower buds and flowers under SD (12L/12D) conditions in the cultivar Harosoy, with the transcription of *GmAP1a* being the most prominent; however, the transcription of *GmAP1d* was not detected in any tissues (Figure 2A). Two *LFY* homologs, designated as *GmLFY1* (Glyma04g37900) [46] and *GmLFY2* (Glyma06g17170), were also identified in the soybean genome. *GmLFY1* was transcribed mainly in developing pods and seeds and was not detected in leaves or the SAM (shoot apex meristem) (Figure 2A), suggesting that the gene might contribute to seed development in soybean instead of flowering, as was previously reported [46]. Similarly to the *AP1* homologs, *GmLFY2* was also transcribed in shoot apices, flower buds and flowers (Figure 2A). Two soybean *SOC1* homologs, *GmSOC1a* (Glyma18g45780) [39] and *GmSOC1b* (Glyma09g40230) [38], could be identified from the soybean genome. Both of these genes were highly expressed in shoot apices, leaves, flower buds and roots but were weakly expressed in flowers and pods (Figure 2A), in agreement with the expression patterns observed for *SOC1* in multiple organs of Arabidopsis [47]. In total, six floral meristem identity genes, *GmAP1a, GmAP1b, GmAP1c, GmSOC1a, GmSOC1b* and *GmLFY2*, were constantly expressed in the shoot apices of the cultivar Harosoy from 10 DAE to 25 DAE under SD (12L/12D) conditions before the floral bud formation stage (floral buds formed at 25 DAE), and *GmSOC1a* and *GmSOC1b* were also constantly expressed in the leaves of the cultivar (Figure 2B). The constantly high expression levels of these genes in shoot apices before the floral bud formation stage most likely indicate their involvement in the flowering transition of soybean.

**Figure 3:**
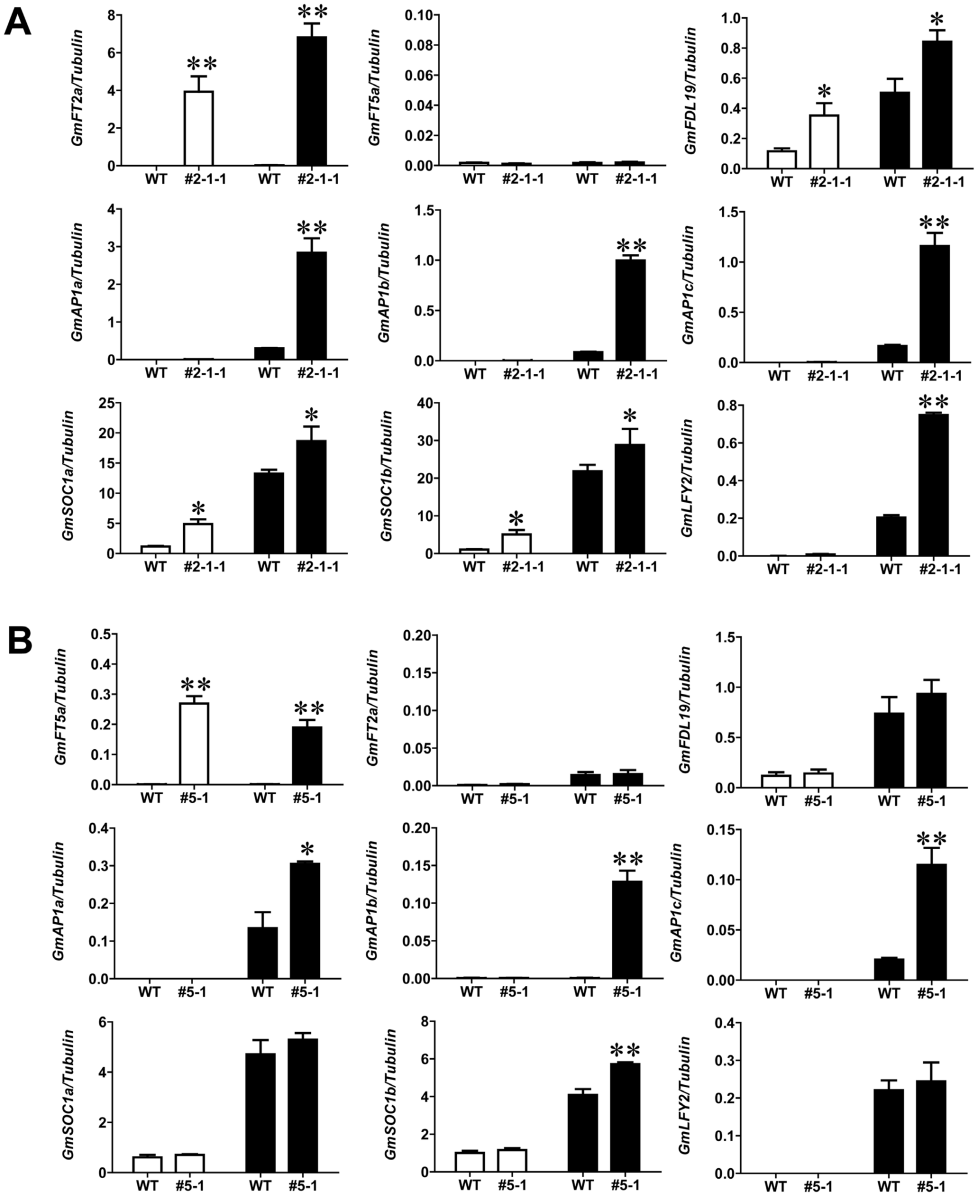
*GmFT2a* and *GmFT5a* promote the expression of soybean flowering-related genes. (A) Expression analyses of *GmFT2a*, *GmFT5a* and flowering-related genes in transgenic *GmFT2a* plants (#2-1-1) and wild-type Williams 82 plants (WT). (B) Expression analyses of *GmFT5a*, *GmFT2a* and flowering-related genes in transgenic *GmFT5a* plants (#5-1) and wild-type Williams 82 plants (WT). The white and black columns represent relative expression in leaves and shoot apices, respectively. Asterisks and double asterisks indicate significant differences between transgenic and WT plants at 0.01<*P*<0.05 and *P*<0.01, respectively.

In Arabidopsis, FT controls photoperiod-regulated flowering by activating the downstream flower meristem identity genes *AP1*, *LFY* and *SOC1* [12]–[14], [48]–[50]. To determine whether GmFT2a and GmFT5a induce early flowering in soybean by regulating the orthologs of *AP1*, *LFY* and *SOC1*, the expression levels of *GmAP1* (*a*, *b*, *c*), *GmSOC1a*, *GmSOC1b* and *GmLFY2* were determined using quantitative PCR in the leaves and shoot apices of the transgenic *GmFT2a* and *GmFT5a* overexpression soybean lines. In the transgenic *GmFT2a* soybean line #2-1-1, the expression levels of *GmFT2a, GmAP1* (*a*, *b*, *c*), *GmSOC1a*, *GmSOC1b* and *GmLFY2* were significantly higher in the shoot apices than were levels in untransformed Williams 82 (Figure 3A), while the expression of *GmFT5a* remained unchanged. However, in the transgenic *GmFT5a* soybean line #5-1, the expression levels of *GmFT5a, GmAP1* (*a*, *b*, *c*), and *GmSOC1b* were significantly higher in the shoot apices than were levels in untransformed Williams 82 (Figure 3B), while the expression levels of *GmFT2a*, *GmSOC1a* and *GmLFY2* were unchanged (Figure 3B). These results suggest that the flowering genes *GmAP1*s, *GmSOC1a*, *GmSOC1b* and *GmLFY2* are downstream of *GmFT2a* and *GmFT5a* and that these genes are most likely differentially involved in the GmFT2a and GmFT5a–induced early flowering of soybean. In addition, the expression of *GmFT2a* in the *GmFT5a* transgenic line and the expression of *GmFT5a* in the *GmFT2a* transgenic line were both unchanged in the leaves and shot apices, suggesting that *GmFT2a* and *GmFT5a* promote soybean flowering in a redundant manner (Figure 3).

### GmFT2a and GmFT5a interact with GmFDL19

**Figure 4:**
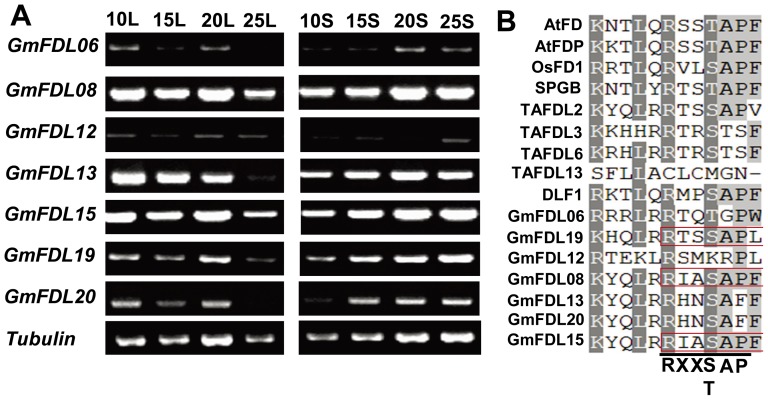
Seven *GmFDLs* (soybean *FD*-like genes) transcribed in leaves and shoot apices. (A) Transcript levels of seven *GmFDLs* in leaves and shoot apices of the soybean cultivar Harosoy under SD (12L/12D) conditions; *Tubulin* is included as an endogenous control. Samples were collected from 10 DAE to 25 DAE. L: leaves; S: shoot apices. (B) Multiple alignment of the amino acid sequences in the SAP motif region of the FDs from soybean and other species. The SAP motif is a putative sequence for FT binding.

FT and its homologs are widely understood to move from leaves to shoot apices, where they interact with FDs to form FT/FD complexes that bind to the promoter of *AP1* [13], [18], [19]. In this study, we found that GmFT2a and GmFT5a promote flowering by inducing the expression of *GmAP1s*, *GmSCO1s* and *GmLFY2*, and it is easily assumed that GmFTs also require a partner such as GmFD to regulate the downstream flowering genes in soybean. Taking the amino acid sequence of FD from Arabidopsis as the query, we searched for orthologs of FD in the soybean genome using Phytozome and identified 18 high-scoring candidate GmFDLs (GmFD-Like) genes (Figure S1). Expression analyses were conducted using RT-PCR for all eighteen selected *GmFDLs*, of which seven *GmFDLs* were transcribed both in leaves and shoot apices (Figure 4A). A multiple sequence alignment of the seven soybean FD-like proteins with the FDs from other species shows a conserved bZIP domain of 42 amino acids (N-X7-R-X9-L-X6-L-X6-L) (Figure S2) and an SAP (RXXS/TAP) motif (Figure 4B), SAP motif has been reported as a putative binding sequence for FT [13]. Phylogenetic analysis was conducted based on the amino acid sequences of the 18 candidate GmFDLs, and the proteins were grouped into three clades (Figure S1, Table S7). GmFDL02, GmFDL04 and GmFDL0602 were divided into the FD clade, but these three GmFDLs did not transcribe or share an SAP motif. GmFDL08, GmFDL13, GmFDL15, GmFDL19 and GmFDL20 were divided into the wheat TaFDL2 clade, with GmFDL08, GmFDL15 and GmFDL19 sharing the classic SAP motif; these three GmFDLs were therefore tested for interactions with soybean GmFT2a and GmFT5a. GmFDL06 and GmFDL12 were divided into the AREB and ABI5 cluster; these two proteins may represent the stress-related bZIP transcription factors in soybean.

**Figure 5:**
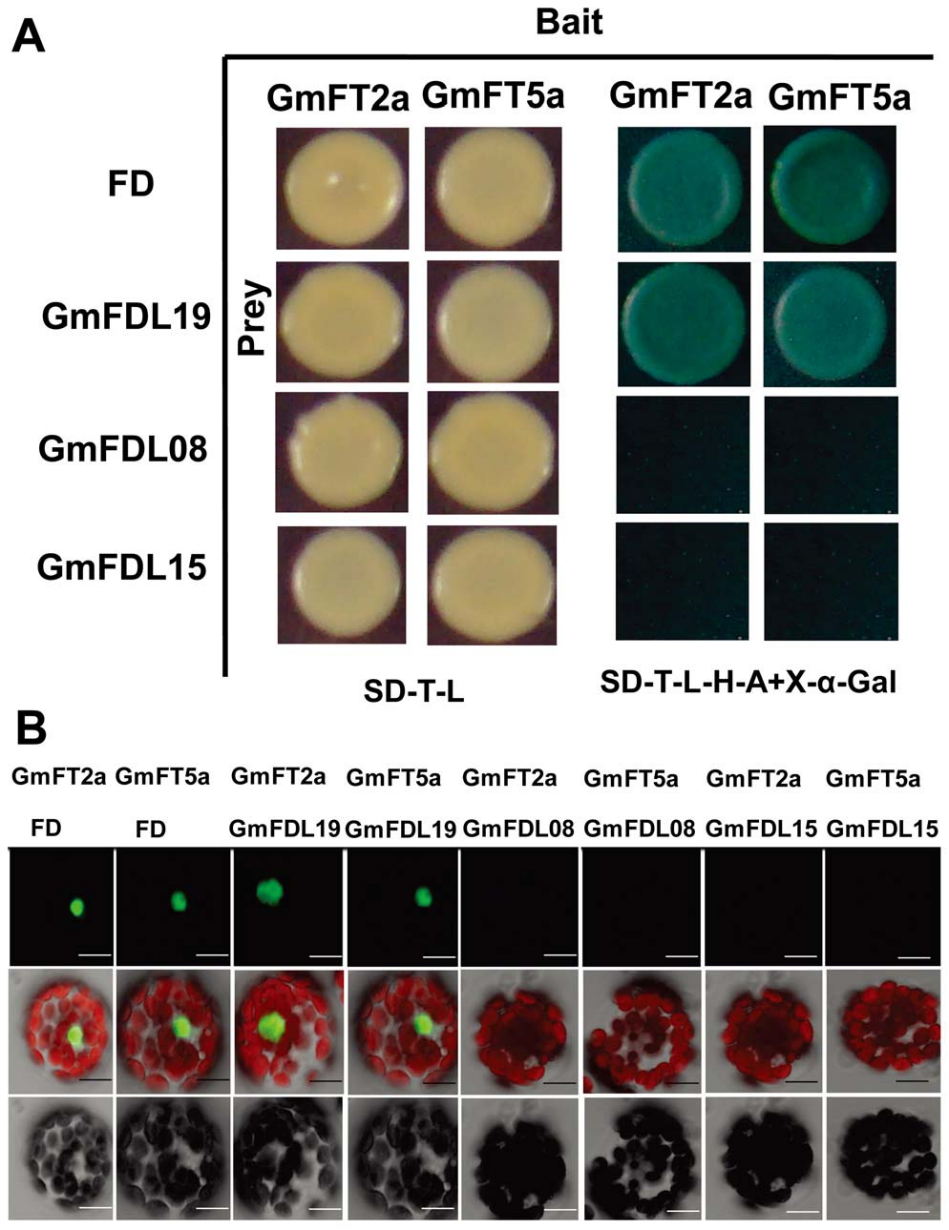
Interactions of GmFDLs with GmFT2a and GmFT5a. (A) Yeast two-hybridization assays; FD was also included because both GmFT2a and GmFT5a promoted early flowering in Arabidopsis. After cotransformation of the baits and preys, an equal amount of yeast clones were plated on SD-Leu-Trp and SD-Leu-Trp-His-Ade+X-α-gal selective plates, and the plates were incubated at 30°C until the emergence of the yeast clones. (B) BiFC (bimolecular fluorescence complementation) assays to confirm the results of the yeast two-hybridization assays. Arabidopsis protoplasts cotransformed with constructs of FTs or FDs fused to the N-terminal (YN) and C-terminal (YC) halves of YFP, respectively (as indicated), were imaged using a confocal microscope after incubation at room temperature (20°C to 25°C) over 18 hours. Images are shown as YFP, merged YFP and bright field. Scale bars indicate 20 µm.

GmFT2a and GmFT5a promoted early flowering in Arabidopsis [35], [36], so we first examined the interactions of FD with GmFT2a and GmFT5a using the yeast two-hybridization assay. The results indicated that both GmFT2a and GmFT5a were able to interact with FD (Figure 5A). Autoactivation tests of GmFT2a and GmFT5a in yeast confirmed that both proteins were unable to activate the reporter genes when used alone as bait (data not presented). The yeast two-hybridization assays of the three SAP motif proteins among the GmFDLs, GmFDL08, GmFDL15 and GmFDL19, with GmFT2a and GmFT5a revealed that only GmFDL19 was able to interact with GmFT2a and GmFT5a (Figure 5A). To validate the results of the yeast two-hybridization tests, *in vivo* BiFC analyses were conducted. These results confirmed that both FD and GmFDL19 could interact with GmFT2a and GmFT5a in the nuclei of Arabidopsis protoplasts (Figure 5B). Our results suggest that GmFT2a and GmFT5a promote early flowering in soybean, most likely through interacting with GmFDL19 and upregulating downstream floral identity genes in a manner similar to that of FT in Arabidopsis. *GmFDL19* was constantly transcribed both in leaves and shoot apices, with increasing expression levels in the shoot apices following the growth stage from 10 DAE to 25 DAE before floral bud formation (25 DAE) (Figure 4A). In addition, the expression of *GmFDL19* was upregulated by GmFT2a in the transgenic overexpression line #2-1-1, while the transcription of *GmFDL19* was unchanged in the transgenic GmFT5a overexpression line #5-1 (Figure 3A, B). The expression patterns and protein interactions of GmFDL19 strongly support this protein as the candidate soybean FD ortholog, which may participate differentially in the early flowering of soybean promoted by GmFT2a and GmFT5a. That is, GmFT2a promotes soybean early flowering through both transcriptional upregulation of and physical interaction with GmFDL19, but GmFT5a only promotes flowering through physical interaction with the protein.

### Overexpression of *GmFDL19* causes early flowering in soybean

**Figure 6:**
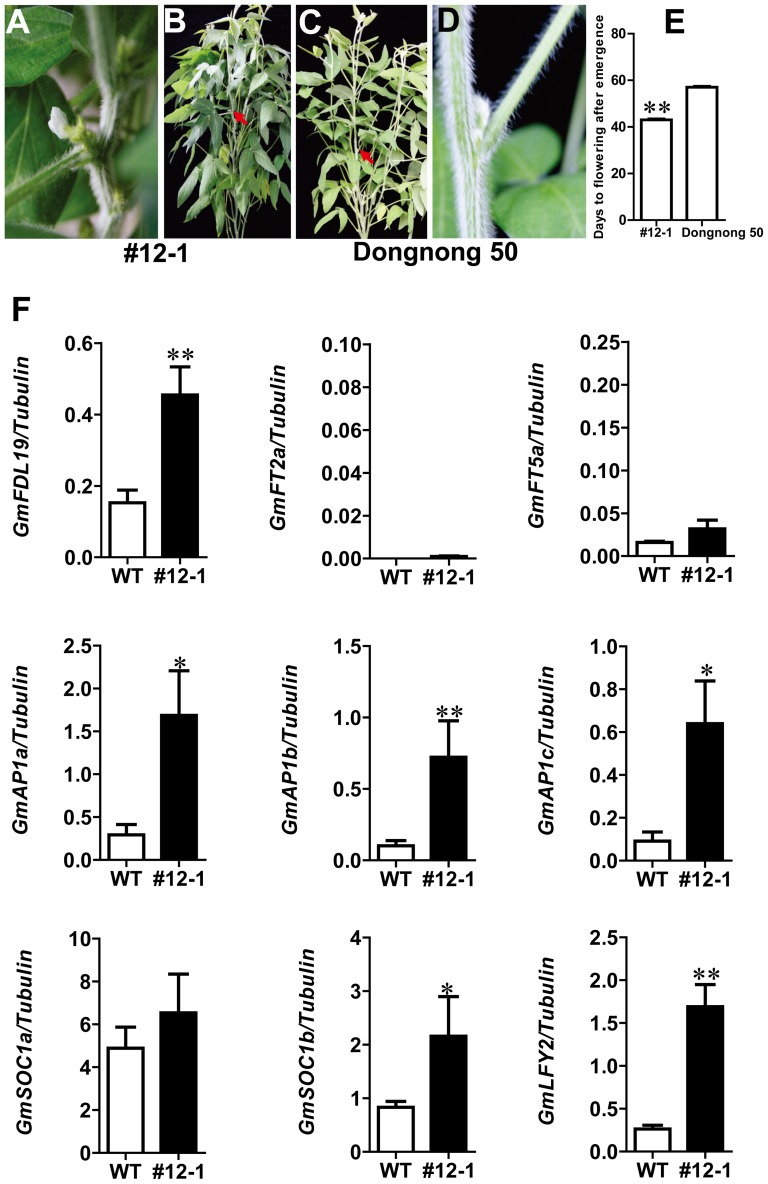
Overexpression of *GmFDL19* in the soybean cultivar Dongnong 50 causes early flowering. (A) The close shot of the transgenic plant in (B) shows the precocious flowers at the axils of the trifoliate leaves (B) A transgenic *GmFDL19* plant showing precocious flowering at the axils of the trifoliate leaves. (C) A wild-type Dongnong 50 plant. (D) The close shot of the wild-type Dongnong 50 plant in (C) does not show flowers at the axils of the trifoliate leaves. (E) Number of days to flowering in transgenic and wild-type plants. Averages and standard errors are calculated from five independent T2 plants and five Dongnong 50 plants. (F) Expression analyses of *GmFDL19* and flowering-related genes in transgenic *GmFDL19* plants (#12-1) and wild-type Dongnong 50 plants (WT); because these flowering related genes are transcribed mostly in shoot apices, shoot apex samples were collected from transgenic and wild-type Dongnong 50 plants at 40 DAE under LD (18L/6D) conditions. Asterisks and double asterisks indicate significant differences from the corresponding wild-type Dongnong 50 at 0.01<*P*<0.05 and *P*<0.01, respectively.

The expression patterns and protein interactions of GmFDL19 suggest that the protein might be involved in the GmFT2a- and GmFT5a-regulated flowering pathway in soybean. To determine the functions of GmFDL19 in soybean flowering, overexpression of *GmFDL19* driven by the 35S promoter was genetically transformed into the cultivar Dongnong 50 (Figure 6A, B). Under LD (16L/8D) conditions, this cultivar flowered early, at approximately 30 DAE. To observe clearer flowering differences between the transgenic T2 line #12-1 and untransformed Dongnong 50, we evaluated the flowering times of both lines under longer photoperiod LD (18L/6D) conditions. Under these conditions, the transgenic line #12-1 flowered, on average, at approximately 43 DAE, while Dongnong 50 flowered at 55 DAE, indicating that GmFDL19 is able to promote flowering in soybean (Figure 6A, B, C, D, E).

We have demonstrated that GmFT2a and GmFT5a promote flowering by inducing the expression of flowering-related genes and that *GmFDL19* transgenic plants flower earlier than untransformed Dongnong 50 plants under an LD photoperiod. We next wished to determine whether these flowering-related genes were also upregulated in the shoot apices of *GmFDL19* transgenic plants. As expected, the transcription levels of *GmFDL19*, as well as those of the flowering related genes *GmAP1s, GmSOC1s* and *GmLFY2*, were significantly higher in the shoot apices of the transgenic *GmFDL19* line #12-1 than in those of untransformed Dongnong 50 (Figure 6F). However, the expression levels of *GmFT2a* and *GmFT5a* in the transgenic *GmFDL19* line #12-1 were unchanged and were only faintly detected in both #12-1 and Dongnong 50 under the LD (18L/6D) conditions (Figure 6F). Considering the upregulation of *GmFDL19* by GmFT2a, as well as the fact that GmFDL19 has the same effect on the upregulation of *GmAP1s, GmSOC1s* and *GmLFY2* as do GmFT2a, the results suggest that *GmFDL19* may act downstream of GmFT2a in the regulation of the flowering transition in soybean. In addition to the transcriptional upregulation of *GmFDL19* by GmFT2a, GmFDL19 interacts with both GmFT2a and GmFT5a, suggesting that GmFDL19 may be required for GmFT2a- and GmFT5a-regulated flowering in soybean.

### GmFDL19 binds to the *GmAP1a* promoter *in vitro*

**Figure 7:**
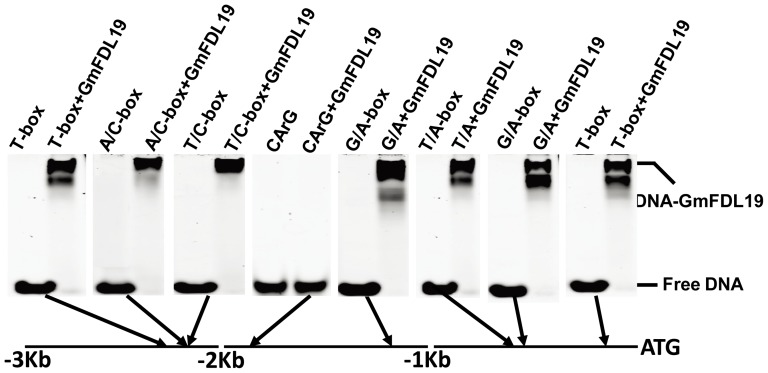
The GmFDL19 protein specifically binds with the ACGT core sequence *in vitro*. Potential bZIP binding sites presented in the *GmAP1a* promoter were used as probes in binding reactions with the purified recombinant GmFDL19 protein. The eight probes included seven potential bZIP binding sites and a CArG-box as negative control. (1) T-box (AACGTT), (2) A/C hybrid of A and C-box (TACGTC), (3) T/C hybrid of T and C-box (AACGTC), (4) CArG-box (CCNNNNNNNNGG), (5) G/A hybrid of G and A-box (CACGTA), (6) T/A hybrid of T and A-box (AACGTA), (7) G/A hybrid of G and A-box (CACGTA), (8) T-box (AACGTT). The scheme below indicates the positions of the various bZIP binding sites.

FT-like proteins interact with FD-like proteins to form FT/FD complexes, which bind to the core ACGT cis-elements located at the promoters of *AP1*-like genes in Arabidopsis, rice and wheat [14], [18], [19]. To determine whether this mechanism is conserved in soybean, we conducted an electrophoretic mobility shift assay (EMSA) to test the binding of GmFDL19 with the promoter of *GmAP1a*. *GmAP1a* was selected for the binding assay because it showed a higher expression level than did the other two homologs, *GmAP1b* and *GmAP1c* (Figure 2, Figure 3 and Figure 6F). This gene contains seven ACGT core elements and one CArG box, representing the putative binding site for MADS domain transcription factors in its promoter (Figure 7). The EMSA results demonstrated that GmFD19 binds to the ACGT core sequences *in vitro*. As the negative control, the CArG box could not be bound by GmFDL19 (Figure 7). These results suggest that the FT/FD-AP1 pathway is well conserved in soybean and that GmFDL19 serves as an important component of GmFT2a- and GmFT5a-regulated flowering in the legume.

## Discussion

### Photoperiod-regulated flowering is integrated through GmFT2a and GmFT5a in soybean

Soybean is adapted to wide range of latitudes, from at least North 50° to South 35°, and its wide adaptability can most likely be attributed to the genetic diversity at many of the major genes (*E1* to *E8* and *J*) and unclassified quantitative trait loci controlling its flowering and maturity. Of the major maturity genes, *E1* to *E4* delay flowering and maturity under LD but have no effects on flowering and maturity under SD [51]. The molecular identities of *E1* to *E4* have recently been characterized [52]. The *E1* gene has the largest effect of the maturity genes on delaying flowering and maturity under LD conditions [29], [33], [51], [53] and has been cloned using a map-based approach, revealing it to be a legume-specific transcription repressor with a putative nuclear localization signal (NLS) and a B3 distantly related domain [29]. The repression of flowering by E1 is most likely due to its suppression of the transcription of *GmFT2a* and *GmFT5a* under LD conditions [29], [36]. *E1* is transcribed mainly in vegetative organs such as cotyledons and leaves [29], which is consistent with the transcription sites of *GmFT2a* and *GmFT5a* [35]. The diurnal circadian rhythm of *E1* transcription contains two peaks in the leaves within 24 hours [29], of which the first transcription peak overlaps with the transcription peaks of *GmFT2a* and *GmFT5a* at 4 hours after dawn [35]. These results suggest that *GmFT2a* and *GmFT5a* are most likely the direct targets of E1 regulation, but this hypothesis requires further evidence for verification. *E3* and *E4* encode the light receptor phytochrome A (PHYA) proteins GmPHYA3 and GmPHYA2, respectively [31], [32]. The expression levels of *GmFT2a* and *GmFT5a* were additively suppressed by E3 and E4 [35], perhaps indirectly via E1 function, as shown in genetic studies revealing that *E3* and *E4* have epistatic effects on *E1* under LD conditions [29]. *E2* was identified molecularly as an ortholog of the Arabidopsis *GIGANTEA* gene [30]. The *E2* gene mainly controls flowering time through the regulation of *GmFT2a*, not *GmFT5a* [30]. Taken together, these results indicate that the photoperiod-regulated flowering pathway in soybean converges at GmFT2a and GmFT5a.

### GmFT2a and GmFT5a redundantly and differentially regulate flowering in soybean

An SD-to-LD transfer experiment demonstrated the differences in photoperiod response between *GmFT2a* and *GmFT5a*. The expression of *GmFT2a* was strictly regulated by photoperiodic changes from SD to LD, whereas the response of *GmFT5a* to photoperiodic changes was gradual, and its expression was maintained at low levels even after the plants were transferred to LD [35]. These findings suggest that, in addition to the phyA-mediated photoperiod response, a second regulatory mechanism may be involved in the differences of expression pattern between *GmFT2a* and *GmFT5a*. In addition to *E3* and *E4*, *E2* influences the mRNA abundance of *FT* homologs. Watanabe et al. (2011) found a clear association between flowering time and *GmFT2a* expression in two sets of near isogenic lines (NILs) for the *E2* locus [30]; dysfunctional *e2* alleles promoted *GmFT2a* expression and conditioned earlier flowering. However, these authors did not observe significant differences in *GmFT5a* expression between the NILs. These results suggest that the *E2* gene (*GmGIa*) mainly controls flowering time through the regulation of *GmFT2a* [30]. The different responses to photoperiodic changes observed between *GmFT2a* and *GmFT5a* [35] may thus be caused by the involvement of the GI (E2)-regulated pathway in *GmFT2a* expression, but not in *GmFT5a* expression. Under the phyA (E3 and E4)-mediated photoperiodic regulation system, GmFT2a and GmFT5a may redundantly and strongly induce flowering under shorter day lengths, but GmFT5a alone may promote flowering in a photoperiod-independent manner under longer day lengths.

The expression analyses of *GmFT2a* and *GmFT5a* in their respective transgenic overexpression lines further demonstrate that GmFT2a and GmFT5a are not regulated by each other, suggesting that GmFT2a and GmFT5a induce soybean flowering redundantly (Figure 3A, B). GmFT2a significantly upregulates downstream floral identity genes such as *GmAP1* (*a*, *b*, *c*), *GmSOC1a*, *GmSOC1b* and *GmLFY2*. However, GmFT5a only upregulates *GmAP1* (*a*, *b*, *c*) and *GmSOC1b* and has no effect on *GmSOC1a* and *GmLFY2* (Figure 3A, B). In addition, the expression of *GmFDL19* was upregulated by GmFT2a in the transgenic overexpression line #2-1-1 while the transcription of the gene was unchanged in the transgenic GmFT5a overexpression line #5-1 (Figure 3A, B). A hypothesis for the system was developed: GmFT2a, in combination with GmFDL19, triggers the upregulation of *GmLFY2*, and *GmLFY2* then feeds back directly to further upregulate *GmFDL19*. However, this hypothesis requires further confirmation. These results suggest that GmFT2a and GmFT5a induce soybean flowering differentially and redundantly. The GmFT2a-regulated flowering pathway and the GmFT5a-regulated flowering pathway may be integrated in the SAM and are redundantly balanced in a very complex manner to ensure precise flowering in paleopolyploid soybean. These two FT homologs may therefore coordinately and redundantly control flowering in soybean.

### GmFDL19 may be involved in GmFT2a- and GmFT5a-regulated flowering in soybean

FT and its homologs are widely known to move from leaves to shoot apices, where they interact with FDs to form FT/FD complexes that bind to the promoter of *AP1* and induce flowering in many plant species [13], [18], [19]. In this study, we report that the bZIP transcription factor GmFDL19 is able to physically interact with two soybean FT homologs, GmFT2a and GmFT5a, as confirmed by both yeast two-hybridization *in vitro* and BiFC *in vivo*. The binding of GmFDL19 with the cis-elements in the promoter of the *AP1* soybean ortholog *GmAP1a* was further confirmed by EMSA *in vitro*. Our results further extend the regulatory module of FT/FD-AP1 in the legume species soybean. In addition to the interaction of GmFDL19 with GmFT2a and GmFT5a, GmFT2a is able to upregulate the transcription of *GmFDL19* in shoot apices. The transcription levels of GmFT2a and GmFT5a are not regulated by GmFDL19, suggesting that GmFDL19 functions downstream of GmFT2a and GmFT5a. The floral identity genes *GmAP1* (*a*, *b*, *c*), *GmSOC1a*, *GmSOC1b* and *GmLFY2* are upregulated by GmFDL19, GmFT2a and GmFT5a in their respective transgenic overexpression lines. Taken together, these results suggest that GmFDL19 may be involved in GmFT2a- and GmFT5a-regulated flowering in soybean.

**Figure 8:**
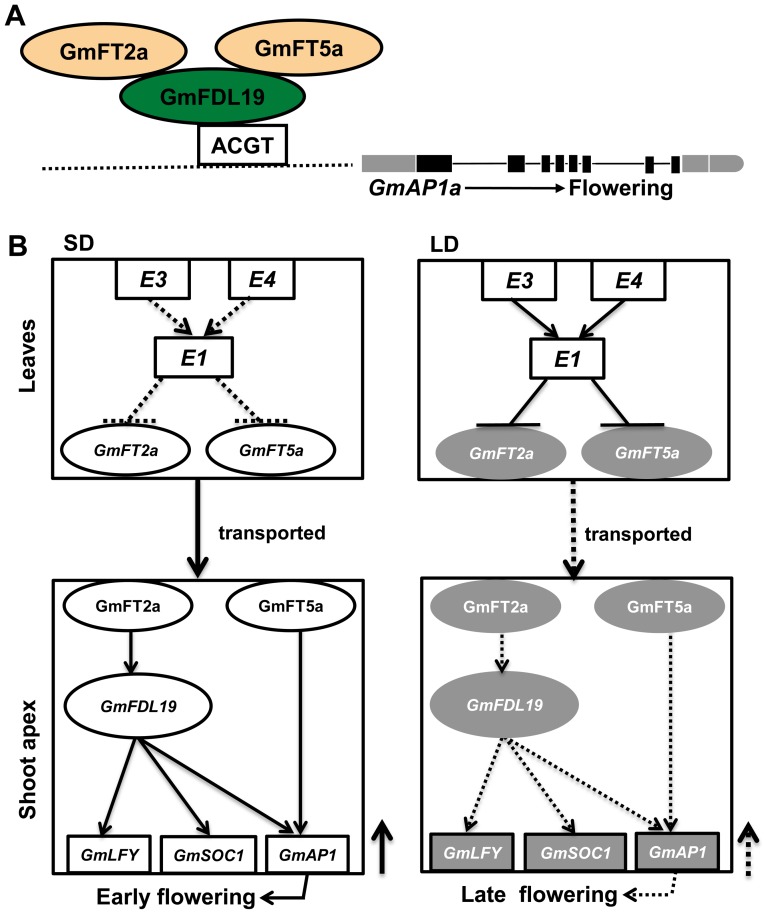
Proposed mechanism of photoperiod-regulated flowering controlled by GmFT2a and GmFT5a in soybean. (A) Model of GmFT2a and GmFT5a regulating the expression of *GmAP1a*. The horizontal dotted line represents the *GmAP1a* promoter, and the black vertical bars indicate the eight exons of *GmAP1a.* The green oval represents the GmFDL19 protein, and this protein can bind to the T-box, G-box or hybrid box (white rectangle) in the *GmAP1a* promoter. The orange oval represents the interactions of GmFT2a and GmFT5a with GmFDL19. (B) A proposed molecular network for photoperiod-regulated flowering in soybean.

In summary, we propose a molecular network of photoperiod-regulated flowering in soybean (Figure 8). Under SD conditions, the *E3, E4* and *E1* genes do not express in leaves where *GmFT2a* and *GmFT5a* are able to transcribe at high levels, and the GmFT2a and GmFT5a proteins are then transported from the leaves to the shoot apices, where they bind with GmFDL19 to induce the expression of flowering-related genes (*GmAP1*, *GmSOC1*, *GmLFY*), thus leading to early flowering. Under LD conditions, *E3* and *E4* genes are highly transcribed in leaves, where they epistatically induce the high expression of the *E1* gene, thereby suppressing the expression levels of *GmFT2a* and *GmFT5a*. The expression levels of flowering related genes are not upregulated due to lack of FT proteins, leading to a late-flowering phenotype.

## Supporting Information

Figure S1 **Phylogenetic relationship of soybean FD-like proteins and FDs from other species constructed using the neighbor-joining method with the program CLUSTAL W.** Bootstrap percentage supports are indicated at the branches of the tree. The seven red filled rectangles indicate the bZIP domain of seven expressed *FD-*like genes in soybean, and the red rectangles indicated the SAP motif contained in soybean FD-like proteins and FDs from other species. The locus IDs or accession numbers of these FDs are presented in Table S7.
(TIF)

Figure S2 **Conserved bZIP domain of the seven soybean FD-like proteins and FDs from other species.**
(TIF)

Table S1 **Primers for qRT-PCR analysis.**
(PDF)

Table S2 **Primers for RT-PCR analysis.**
(PDF)

Table S3 **Primers for isolation of seven** ***GmFDLs*** **.**
(PDF)

Table S4 **Primers for yeast two-hybridization assays.**
(PDF)

Table S5 **Primers for BiFC.**
(PDF)

Table S6 **Primers for EMSA.**
(PDF)

Table S7 **List of** ***FD*** **homologs contained in the phylogenetic analysis used in the present study.**
(PDF)
