# Supplementary figures and images for: GmFT2a and GmFT5a Redundantly and Differentially Regulate Flowering through Interaction with and Upregulation of the bZIP Transcription Factor GmFDL19 in Soybean
Source: PLoS One. 2014 May 20;9(5):e97669. doi: 10.1371/journal.pone.0097669 (PMC4028237; doi:10.1371/journal.pone.0097669)

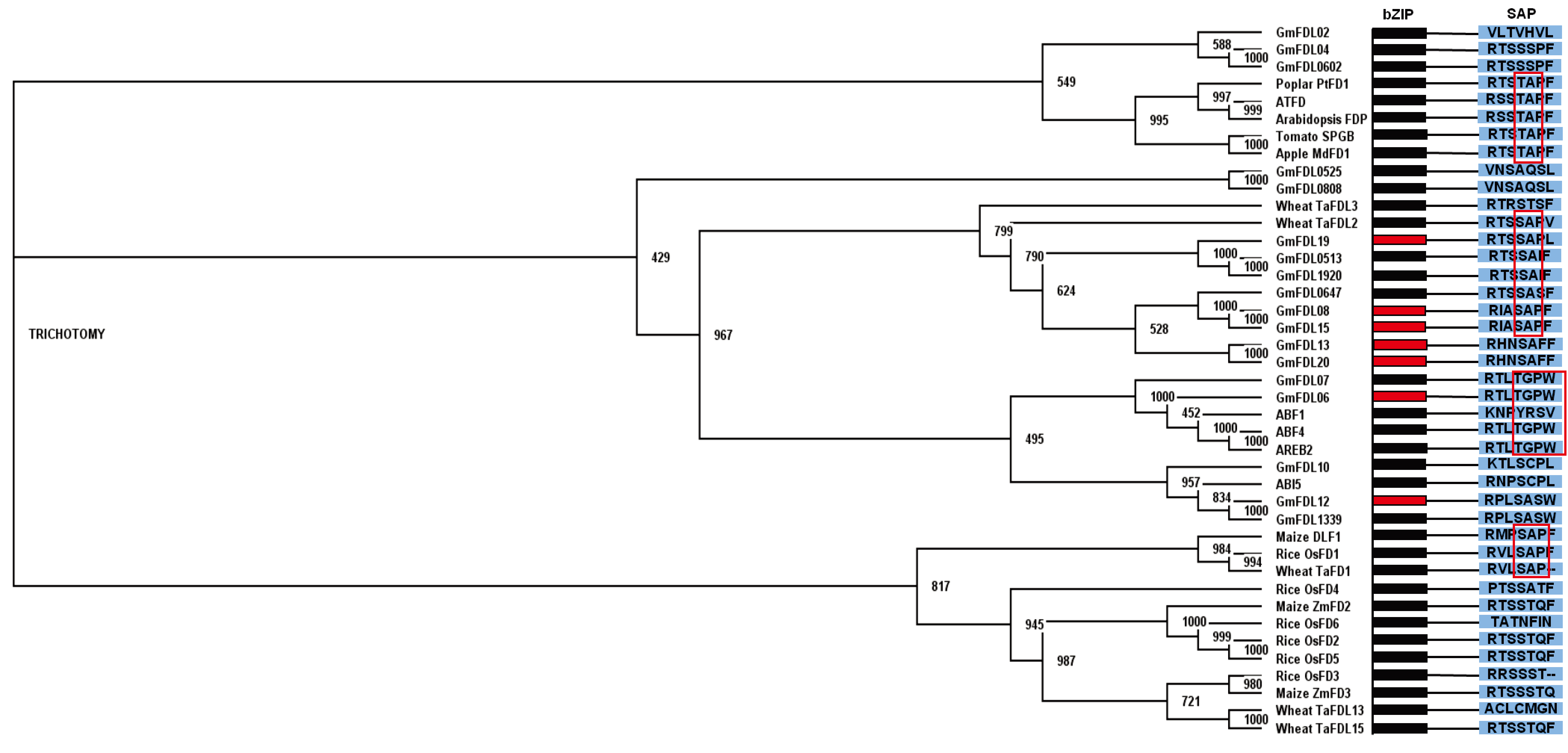

Supplement: Figure S1 — Phylogenetic relationship of soybean FD-like proteins and FDs from other species constructed using the neighbor-joining method with the program CLUSTAL W. Bootstrap percentage supports are indicated at the branches of the tree. The seven red filled rectangles indicate the bZIP domain of seven expressed FD-like genes in soybean, and the red rectangles indicated the SAP motif contained in soybean FD-like proteins and FDs from other species. The locus IDs or accession numbers of these FDs are presented in Table S7. (TIF) [file pone.0097669.s001.tif]

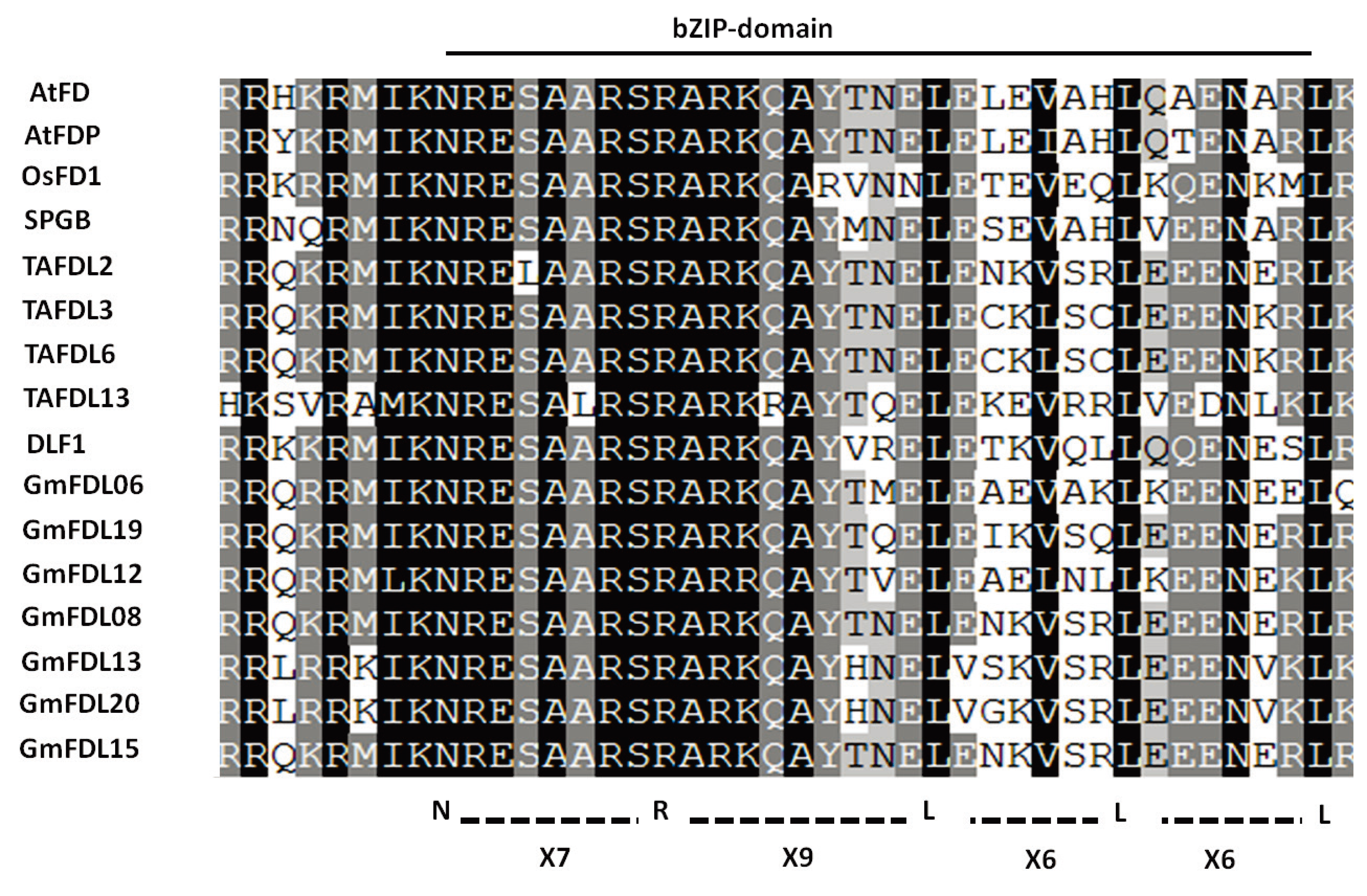

Supplement: Figure S2 — Conserved bZIP domain of the seven soybean FD-like proteins and FDs from other species. (TIF) [file pone.0097669.s002.tif]
