# Supplementary material for: GmFT2a and GmFT5a Redundantly and Differentially Regulate Flowering through Interaction with and Upregulation of the bZIP Transcription Factor GmFDL19 in Soybean
Source: PLoS One. 2014 May 20;9(5):e97669. doi: 10.1371/journal.pone.0097669 (PMC4028237; doi:10.1371/journal.pone.0097669)
Supplement: Table S1 — Primers for qRT-PCR analysis. (PDF) [file pone.0097669.s003.pdf]

Table S1. Primers for qRT-PCR analysis

Target gene	Primer name	Primer sequence (5'-3')
GmFT2a	*GmFT2a*-qF1	GGATTGCCAGTTGCTGCTGT
	GmFT2a-qR1	GAGTGTGGGAGATTGCCAAT
GmFT5a	*GmFT5a*-qF1	GCCTTACTCCAGCTTATACT
	GmFT5a-qR1	GGCATGCTCTAGCATTGCAA
GmAP1a	*GmAP1a*-qF1	TGAACATGGGTGGCAATTAC
	GmAP1a-qR1	TGTCAAATGCCATACCAAAG
GmAP1b	*GmAP1b*-qF1	TAGTTTGGCTGCTCCCATTG
	GmAP1b-qR1	GGAATGATCTCATGTACGCT
GmAP1c	*GmAP1c*-qF1	GAAAGAAAAGGTTGCAGCTTC
	GmAP1c-qR1	GCATCCAAGGTGACAGGAAT
GmAP1d	*GmAP1d*-qF1	TAGTTTGGCTGCTCCCATTG
	GmAP1d-qR1	GGAATGATCTCATGTACGAG
GmSOC1a	*GmSOC1a*-qF1	CGAGTTGCTTTTTTTCCCTAG
	GmSOC1a-qR1	TGAGTCTTTCCTCTCACCAT
GmSOC1b	*GmSOC1b*-qF1	CTTTGCTAGCTACCCTCTCT
	GmSOC1b-qR1	TGAGTCTTTCCTCTCACCAT
GmLFY1	*GmLFY1*-qF1	CTGAGAGAAACAGTGTCTCG
	GmLFY1-qR1	AGCTAGCAAGGTTCTACACA
GmLFY2	*GmLFY2*-qF1	CTGAGAGAAACAGTGCCGCA
	GmLFY2-qR1	AGCTAGCAAGGTTCTACACA
GmFDL19	*GmFDL19*-qF1	GGTTTGGAGAGATATGCAAC
	GmFDL19-qR1	GGCATGTTGTGATGTGTTGT
Tubulin	*Tub*-F2	GAGAAGAGTATCCGGATAGG
	Tub-R1	GTTTCCGAACACTCAAGCTC
