# Supplementary material for: GmFT2a and GmFT5a Redundantly and Differentially Regulate Flowering through Interaction with and Upregulation of the bZIP Transcription Factor GmFDL19 in Soybean
Source: PLoS One. 2014 May 20;9(5):e97669. doi: 10.1371/journal.pone.0097669 (PMC4028237; doi:10.1371/journal.pone.0097669)
Supplement: Table S2 — Primers for RT-PCR analysis. (PDF) [file pone.0097669.s004.pdf]

Table S2. Primers of RT-PCR analysis

Target gene	Primer name	Primer sequence (5'-3')
GmFDL02	*GmFDL02*-F1	ACACCAAACCCCAATAAG
	GmFDL02-R1	ACCAAATGAGGGAAAAGA
GmFDL04	*GmFDL04*-F1	ATGGCCTCATCGCCATGTGA
	GmFDL04-R1	CATCCAAGAGTCATGATGGG
GmFDL0513	*GmFDL0513*-F1	ATGAGATAGAGAGGGCATTG
	GmFDL0513-R1	TAATTCCCACAAATGGATCCG
GmFDL0525	*GmFDL0525*-F1	ATGGCGTCGTCGAAGCTTGT
	GmFDL0525-R1	GCTCTTCCTTTAATAACCG
GmFDL06	*GmFDL06*-F1	TGGATCAGGTTGAGATTGCG
	GmFDL06-R1	AAACTCCTCCAACGTCAT
GmFDL0602	*GmFDL0602*-F1	ATGGCCTCATCACCATGTGA
	GmFDL0602-R1	TCAAAATGGAGATGAAGAGG
GmFDL0647	*GmFDL0647*-F1	AGGAGCTAGAGAAAATGTTG
	GmFDL0647-R1	GCCACTGGTAATAATCTGC
GmFDL07	*GmFDL07*-F1	ATGAACTTCAGGAACTTTGG
	GmFDL07-R1	GAACCTTGTCTTTGCAAACC
GmFDL08	*GmFDL08*-F1	ATGGGGACCCAAACTATG
	GmFDL08-R1	CCACACAGTAATCCAGAAGG
GmFDL0808	*GmFDL0808*-F1	GCAGTTATTAAATGAAGAGGC
	GmFDL0808-R1	CCCAGAACCATGGATCTTCTT
GmFDL10	*GmFDL10*-F1	ATGGTGGTGCCTGAGTCTCAGAT
	GmFDL10-R1	CTAAAGACAAGTTGTTGTGG
GmFDL12	*GmFDL12*-F1	TTGACCCAATGAAGCTAAG
	GmFDL12-R1	GAGACCAAATTTCATCCACA
GmFDL13	*GmFDL13*-F1	GAATGGGGATTCAGACAATG
	GmFDL13-R1	CAATTGAAGGGGAGGACAAC
GmFDL1339	*GmFDL1339*-F1	ATGTCTCTCCAACAACC
	GmFDL1339-R1	AGGGACAGATAATGAGCCTG
GmFDL15	*GmFDL15*-F1	ATGGGGACCCAAGGCAAAAC
	GmFDL15-R1	CCACACAGTAATCCAGAAGG
GmFDL19	*GmFDL19*-F1	ATGAGATAGAGAGGGCATTG
	GmFDL19-R1	TGCCGACCTTTAAAGCACGG
GmFDL1920	*GmFDL1920*-F1	ATGAGATAGAGAGGGCATTG
	GmFDL1920-R1	TAATTCCCACAAATGGGCCC
GmFDL20	*GmFDL20*-F1	GAATGGGGATTCAGACAGTG
	GmFDL20-R1	ATCATCCTCCCCCTCAATCG
Tubulin	*Tub*-F1	GACCCGATAACTTCGTGTTC
	Tub-R1	GTTTCCGAACACTCAAGCTC
