# Supplementary material for: GmFT2a and GmFT5a Redundantly and Differentially Regulate Flowering through Interaction with and Upregulation of the bZIP Transcription Factor GmFDL19 in Soybean
Source: PLoS One. 2014 May 20;9(5):e97669. doi: 10.1371/journal.pone.0097669 (PMC4028237; doi:10.1371/journal.pone.0097669)
Supplement: Table S3 — Primers for isolation of seven GmFDLs . (PDF) [file pone.0097669.s005.pdf]

Table S3. Primers for seven full-length GmFDLs isolation

Target gene	Primer name	Primer sequence (5'-3')
GmFDL06	*GmFDL06*-QF1	GGCATGAATTTCAAGAGC
	GmFDL06-QR1	CTACCATGGACCAGTTTGTG
GmFDL08	*GmFDL08*-QF1	ATGGGGACCCAAACTATG
	GmFDL08-QR1	ATACAAACCGCTACAACT
GmFDL12	*GmFDL12*-QF1	TCTCTCCAACAACCAAA
	GmFDL12-QR1	CCAGGATGCGCTTAGAG
GmFDL13	*GmFDL13*-QF1	ATGGGGATTCAGACAATGGGA
	GmFDL13-QR1	TGTCCCCATCAAATCA
GmFDL15	*GmFDL15*-QF1	ATGGGGACCCAAAGGCAAAAC
	GmFDL15-QR1	CCGCACAGTAATCCACAAGGAG
GmFDL19	*GmFDL19*-QF1	ATGGGATCTCAAGGTGG
	GmFDL19-QR1	GAGAACTATGGAAGTGCATCA
GmFDL20	*GmFDL20*-QF1	ATGGGGATTCAGACAGTGGGA
	GmFDL20-QR1	ATAACTGCCTAGCCCTAGTAC
