# Supplementary material for: GmFT2a and GmFT5a Redundantly and Differentially Regulate Flowering through Interaction with and Upregulation of the bZIP Transcription Factor GmFDL19 in Soybean
Source: PLoS One. 2014 May 20;9(5):e97669. doi: 10.1371/journal.pone.0097669 (PMC4028237; doi:10.1371/journal.pone.0097669)
Supplement: Table S4 — Primers for yeast two-hybridization assays. (PDF) [file pone.0097669.s006.pdf]

Table S4. Primers for yeast two-hybridization assays

Construct name	Primers sequence (5'-3')	Restriction sites	Function
pGBKT7-*GmFT2a*	CCATGGAGATGCCTAGTGGAAGTAGGGAT	*NcoI*	Bait
	GTCGACGAGTGTGGGAGATTGCCAAT	*SalI*	
pGBKT7-*GmFT5a*	GAATTCATGGCACGGGAGAACCCTCTT	*EcoRI*	Bait
	GTCGACGGCATGCTCTAGCATTGCAA	*SalI*	
pGADT7-*GmFD08*	GAATTCATGGGGACCCAAACTATG	*EcoRI*	Prey
	GGATCCATACAAACCGCTACAACT	*BamHI*	
pGADT7-*GmFD15*	GAATTCATGGGGACCCAAAGGCAAAAC	*EcoRI*	Prey
	GGATCCATACAAACCGCTACAACT	*BamHI*	
pGADT7-*GmFD19*	GCCATATGATGGGATCTCAAGGTGG	*NdeI*	Prey
	ATGGATCCGAGAACTATGGAAGTGCATCA	*BamHI*	
pGADT7-*FD*	GAATTCATGTTGTCATCAGCTAAGC	*EcoRI*	Prey
	GGATCCCAATCCCCAAAAGAGAAACAAG	*BamHI*	

Underline in primer sequence highlights the restriction enzyme recognition site
