# Supplementary material for: GmFT2a and GmFT5a Redundantly and Differentially Regulate Flowering through Interaction with and Upregulation of the bZIP Transcription Factor GmFDL19 in Soybean
Source: PLoS One. 2014 May 20;9(5):e97669. doi: 10.1371/journal.pone.0097669 (PMC4028237; doi:10.1371/journal.pone.0097669)
Supplement: Table S5 — Primers for BiFC. (PDF) [file pone.0097669.s007.pdf]

Table S5. Primers for BiFC

Construct name	Primers sequence (5'-3')	Restriction sites	Function
puc_SPYNE-*GmFT2a*	TCTAGAATGCCTAGTGGAAGTAGGGAT	*XbaI*	BiFC
	GTCGACGTATAACCTCCTTCCACCAGA	*SalI*	
puc_SPYNE-*GmFT5a*	TCTAGA ATGGCACGGGAGAACCCTCTT	*XbaI*	BiFC
	GTCGACATATCTCCTTCCACCGCAAC	*SalI*	
puc_SPYCE-*FD*	TCTAGAATGTTGTCATCAGCTAAGC	*XbaI*	BiFC
	GTCGACAAATGGAGCTGTGGAAGACCG	*SalI*	
puc_SPYCE-*GmFDL19*	TCTAGAATGGGATCTCAAGGTGG	*XbaI*	BiFC
	GTCGACAAGGGGAGCTGAACTAGTTCT	*SalI*	
puc_SPYCE-*GmFDL08*	TCTAGAATGGGGACCCAAACTATG	*XbaI*	BiFC
	GTCGACATACAAACCGCTACAACT	*SalI*	
puc_SPYCE-*GmFDL15*	TCTAGAATGGGGACCCAAAGGCAAAAC	*XbaI*	BiFC
	GTCGACCCGCACAGTAATCCACAAGGAG	*SalI*	

Underline in primer sequence highlights the restriction enzyme recognition site
