# Supplementary material for: GmFT2a and GmFT5a Redundantly and Differentially Regulate Flowering through Interaction with and Upregulation of the bZIP Transcription Factor GmFDL19 in Soybean
Source: PLoS One. 2014 May 20;9(5):e97669. doi: 10.1371/journal.pone.0097669 (PMC4028237; doi:10.1371/journal.pone.0097669)
Supplement: Table S6 — Primers for EMSA. (PDF) [file pone.0097669.s008.pdf]

Table S6. Primers for EMSA

Primer name	Type	Core sequcence	Primers sequence (5'-3')
*GmAP1a*P-732F	TBOX	AACGTT	CCAAAAAATCAAACGTTTAGGTAAAAACCCAAATGATCTA
*GmAP1a*P-732R			TAGATCATTTGGGTTTTTACCTAAACGTTTGATTTTTTGG
*GmAP1a*P-911F	A/C BOX	TACGTC	AACACCTCCTACGTCCATGATATTGCAGCGACCTAAAAAA
*GmAP1a*P-911R			TTTTTTAGGTCGCTGCAATATCATGGACGTAGGAGGTGTT
*GmAP1a*P-944F	T/C BOX	AACGTC	GACCTAAAAAACAAACGTCGGAAGACGAACTTGTAGAAAG
*GmAP1a*P-944R		GACGTT	CTTTCTACAAGTTCGTCTTCCGACGTTTGTTTTTTAGGTC
*GmAP1a*P-1876F	G/A BOX	CACGTA	CGCGTGTACGCGTACCACGTATCCCAATTCCATAAGAAAT
*GmAP1a*P-1876R			ATTTCTTATGGAATTGGGATACGTGGTACGCGTACACGCG
*GmAP1a*P-2471F	T/A	AACGTA	TCGTGGGTCGTGGCGCCGTCAACGTAAGGATGCACGTAAT
*GmAP1a*P-2471R			ATTACGTGCATCCTTACGTTGACGGCGCCACGACCCACGA
*GmAP1a*P-2483F	G/A BOX	CACGTA	GGCGCCGTCAACGTAAGGATGCACGTAATGTTCTGGTTCA
*GmAP1a*P-2483R			TGAACCAGAACATTACGTGCATCCTTACGTTGACGGCGCC
*GmAP1a*P-2703F	TBOX	AACGTT	GATCGACAGCGCAACGTTAAGAAAACTACCCCGGTTTTTC
*GmAP1a*P-2703R			GAAAAACCGGGGTAGTTTTCTTAACGTTGCGCTGTCGATC
*GmAP1a*P-1280F	CArG BOX	CCAAAAATAAGG	GCAACCATCCATCCAAAAATAAGGCAAAAAAAATAAACAT
*GmAP1a*P-1280R			ATGTTTATTTTTTTTGCCTTATTTTTGGATGGATGGTTGC
GmFDL19-29b-F			CATATGATGGGATCTCAAGGTGG
GmFDL19-29b-R			GTCGACGAGAACTATGGAAGTGCATCA

Underline in primer sequence highlights the restriction enzyme recognition site
