# Supplementary material for: GmFT2a and GmFT5a Redundantly and Differentially Regulate Flowering through Interaction with and Upregulation of the bZIP Transcription Factor GmFDL19 in Soybean
Source: PLoS One. 2014 May 20;9(5):e97669. doi: 10.1371/journal.pone.0097669 (PMC4028237; doi:10.1371/journal.pone.0097669)
Supplement: Table S7 — List of FD homologs contained in the phylogenetic analysis used in the present study. (PDF) [file pone.0097669.s009.pdf]

Tables S7. List of FD homologs contained in phylogenetic analysis

Speices	Gene name	Locus ID/Accession number	Database
Glycine max	*GmFDL02*	Glyma02g05100/PKD12088	Phytozome/TGI
Glycine max	*GmFDL04*	Glyma04g02420/TC443698	Phytozome/TGI
Glycine max	*GmFDL0513*	Glyma05g13890/TC437102	
Glycine max	*GmFDL0525*	Glyma05g25200/TC164184	
Glycine max	*GmFDL06*	Glyma06g04350/TC442362/TC455720	
Glycine max	*GmFDL0602*	Glyma06g02470/BI970547/TC435144	
Glycine max	*GmFDL0647*	Glyma06g47220/TC423836	
Glycine max	*GmFDL07*	Glyma07g33600/TC447121	
Glycine max	*GmFDL08*	Glyma08g24340/TC470995	
Glycine max	*GmFDL0808*	Glyma08g08220/TC437419	
Glycine max	*GmFDL10*	Glyma10g08370/TC462107	
Glycine max	*GmFDL12*	Glyma12g30980/TC462817	
Glycine max	*GmFDL13*	Glyma13g03880/GE096639	
Glycine max	*GmFDL1339*	Glyma13g39340/HO032893	
Glycine max	*GmFDL15*	Glyma15g35080/TC445245	
Glycine max	*GmFDL19*	Glyma19g30230/TC469368	
Glycine max	*GmFDL1920*	Glyma19g20090/TC454037	
Glycine max	*GmFDL20*	Glyma20g10060/CO983201	
Arabidopsis thaliana	*FD*	AT4G35900	TAIR
Arabidopsis thaliana	*FDP*	AT2G17770	
Populus trichocarpa	*PtFD1*	POPTR_0005S11140	Phytozome
Solanum lycopersicum	*SPGB*	Solyc02g083520	Sol genomics network
Malus domestica	*MdFD1*	MDP0000169473	Genome database for Rosaceae
Zea mays	*DLF1*	GRMZM2G067921	Maizesequence.org
Triticum aestivum	*TaFD1*	CK206464	Genebank
Triticum aestivum	*TaFDL2*	ABZ91908	
Triticum aestivum	*TaFDL3*	ABZ91909	
Triticum aestivum	*TaFDL13*	ABZ91911	
Triticum aestivum	*TaFDL15*	ABZ91912	
Oryza sativa	*OsFD1*	Os09g0540800	RAP-DB/The MSU Rice Genome Anotation Project Database
Oryza sativa	*OsFD2*	Os06g0720900	
Oryza sativa	*OsFD3*	Os02g0833600	
Oryza sativa	*OsFD4*	Os08g0549600	
Oryza sativa	*OsFD5*	Os06g0724000	
Oryza sativa	*OsFD6*	Os06g0719500	
Arabidopsis thaliana	*ABF1*	AT1G49720	TAIR
Arabidopsis thaliana	*ABF4*	AT3G19290	
Arabidopsis thaliana	*AREB2*	AT3G19290	
Arabidopsis thaliana	*ABI5*	AT2G36270	

Phytozome (http://www.phytozome.net/)

TGI (http://compbio.dfci.harvard.edu/tgi/)

TAIR (http://www.arabidopsis.org/)

sol genomics network (http://solgenomics.net/)

Genome database for Rosaceaehttp (http://www.rosaceae.org/)

Maizesequence.org (http://ensembl.gramene.org/Zea_mays/Info/Index)

Genebank (http://www.ncbi.nlm.nih.gov/)

RAP-DB (http://rapdb.dna.affrc.go.jp/)

The MSU Rice Genome Anotation Project Database (http://rice.plantbiology.msu.edu/)
